# Viability predictive factors of the daughter vesicles in hepatic cystic echinococcosis

**DOI:** 10.1186/s12879-023-08937-y

**Published:** 2024-01-03

**Authors:** Aymen Trigui, Nahed Khmekhem, Sami Fendri, Rahma Daoud, Amira Akrout, Jihene Trabelsi, Rafik Mzali, Fatma Cheikhrouhou, Ali Ayadi, Chadli Dziri, Mohamed Ben Amar, Saleh Boujelbene

**Affiliations:** 1 Faculty of Medicine, Department of General and Digestive Surgery, University of Sfax, Habib Bourguiba Hospital, Sfax, 3029 Tunisia; 2 Faculty of Medicine, Department of Parasitology and Mycology, University of Sfax, Habib Bourguiba Hospital, Sfax, Tunisia; 3 Faculty of Medicine, Department of Epidemiology, University of Sfax, Hedi Cheker Hospital, Sfax, Tunisia; 4 General Surgery, Honoris Medical Simulation Center Director, Medical school of Tunis, University El Manar, Tunis, Tunisia

**Keywords:** Echinococcus Infection, Hepatic, Daughter vesicles

## Abstract

**Introduction:**

Management of cystic echinococcosis (CE) requires knowledge of certain aspects related to the survival of *Echinococcus granulosus*. The viability of daughter vesicles (DV) is a determining factor in guiding therapeutic indications, particularly for transiently active Cysts type CE3b.

**Purpose:**

To determine the predictive factors of DV viability and its impact on the therapeutic management of CE3b type.

**Materials and methods:**

This is a prospective pilot study with an analytical aim on patients with cystic echinococcosis of the liver type CE2 and CE3b, operated in the General Surgery Department of Habib-Bourguiba Academic Hospital, Sfax-Tunisia for 22 months from March 2018 until December 2019. The unit of the study is the DV. A parasitological study of the DV was done in the parasitology laboratory.

**Results:**

During the study period, 27 (40.9%) of 66 operated CE Disease from 21 patients containing 248 DV were explored. The median viability of DV protoscoleces was 16.7%. In bivariate analysis, factors for viability of DV protoscoleces were: fever, acute cholangitis, hyperbilirubinemia, left liver location, rock water and bilious echinococcal fluid (EF), cyst size ≥ 43 mm, Intracystic pressure ≥ 35 mmHg, DV size ≥ 6.5 mm, volume, number of DV/cyst ≥ 5, and opaque wall (*p* < 0.05). Predictive factors for the Non-viability of DV were: CE3b type, purulent EF, gelatinous EF. In multivariate analysis, only CE2 type, cyst size ≥ 43 mm, number of DV/cyst ≥ 5 and DV size ≥ 6.5 mm were factors significantly associated with the viability of DV protoscoleces.

**Conclusion:**

CE3b cysts without the criteria of viability of DV protoscoleces may become candidates for the ‘Wait-and-Watch’ procedure.

## Introduction

Cystic echinococcosis (CE) is a parasitic anthropozoonosis due to the development of the larval form of a cestode, *Echinococcus granulosus* in humans. Proper management of CE requires knowledge of certain aspects related to the survival of the parasitic agent, particularly protoscolex. In the literature, the study of the fertility of cysts and the viability of protoscoleces has always been performed on echinococcal fluid (EF) [1, 2]. However, no study concerning the lesional aspects of daughter vesicles (DV) is available at present. Therefore, a better knowledge of these aspects as well as of the predictive factors of echinococcal dissemination allows us to guide our future practices to guide the therapeutic indications. Indeed, while therapeutic indications are increasingly codified for active cysts (I/ II/ II Gharbi and CE1/CE2/CE3a WHO) and inactive cysts (IV/V Gharbi and CE4/CE5 WHO) [3, 8], cysts of transient activity, especially CE3b (CE3b WHO) are not codified. This type has long been considered a CE4 cyst that contains DV. With this in mind, in the present study, we addressed the parasite content of daughter vesicles in viable protoscolex (Viability of DV protoscoleces) from human liver cystic echinococcosis (LCE) operated on in our Department and the aim of this study was to search for an association with data from patients operated on for LCE, the characteristics of the cysts and DV, and the impact of these parameters on the therapeutic management of LCE, and in particular transiently active cysts of the CE3b type.

## Materials and methods

We conducted a prospective analytical pilot study including patients with cystic echinococcosis of the liver type CE2 and CE3b according to the WHO classification, operated in the General Surgery Department of the Habib Bourguiba Academic Hospital of Sfax-Tunisia, for 22 months from March 1, 2018, until December 31, 2019. We performed a microscopic study of the cyst contents and daughter vesicles.

We included multivesicular cystic echinococcosis of the liver type CE2 and CE3b of the WHO classification [9]. We did not include patients with severe cholangitis or shock and excluded destroyed DV and DV not preserved at + 4 °C. The unit of this study was the DV. The number of daughter vesicles required for this study was determined from data from a pre-investigation performed on 20 daughter vesicles from 5 different liver cystic echinococcosis. Thus, we found a viability of 20%, and the necessary number of DV was estimated to be 246 with a precision of 5%. Before the initiation of our study, an agreement was authorized by the Committee for the Protection of Persons Suitable for Medical or Scientific Experimentation of Medicinal Products for Human Medicine “CPP” under the reference “CPP sud number 0021/2017”. The CPP is the only center for medical or scientific research on medicinal products of human medicine in our institution " The University of Sfax”, approved by the Ministry of Public Health. All patients, who participated in our prospective study, signed a consent agreement after being informed by our research protocol.

The unit of this study was the DV. The primary endpoint was the viability of the daughter vesicle. Viability was defined as the presence of viable protoscolex within the cyst. This viability is equal to the number of viable protoscolex to the total number (or a sample of 150 if the fluid is very fertile) of protoscolex in the EF (2). A pre-established form was filled in with the data of the operated patient, the cyst, the echinococcal fluid (EF) and the daughter vesicles (DV) for which a macroscopic and microscopic study was performed.

The study protocol was standardized: A standard workup and systematic preoperative imaging such as ultrasound and abdominopelvic CT were performed. In the operating room, all patients had an intraoperative ultrasound followed by a systematic measurement of the intracystic pressure. To measure intracystic pressure, a needle puncture is performed and connected to an invasive pressure transducer commonly employed for arterial pressure measurements. Then, the pressure is recorded, and the intracystic pressure measured corresponds to the pressure displayed on the scope. Then, for each cyst, an echinococcal fluid aspiration was performed with a graduated syringe before cystotomy, and the DV were collected with a sterile spoon and placed in a container. The echinococcal material (EF + DV) was sent to the Parasitology-Mycology laboratory on the same day of the surgery or at the latest the next day after conservation at + 4 °C. The daughter vesicles are treated in the laboratory with a standardized technique as well: a random sample is thus taken from a petri dish of 10 intact DV if the cyst contains a number ≥ 10 DV / cyst or all the DV if the number of the latter/cyst is < 10, different in size and appearance using a spoon to avoid selection bias. Then, these vesicles are washed with a sterile 0.9 sodium chloride solution, the volume of each DV is measured with a test tube or a graduated syringe, the liquid of each DV is collected with a sterile syringe and sedimented for 30 min and the blades are read under a light microscope after adding eosin.

For the macroscopic study of the echinococcal material, a visual description of the HF and the DV was performed. We described the puncture fluid (rock water, bilious, purulent, or gelatinous) and for the DV, we studied its consistency (tense or flaccid) and its transparency (opaque or transparent).

**Fig. 1:**
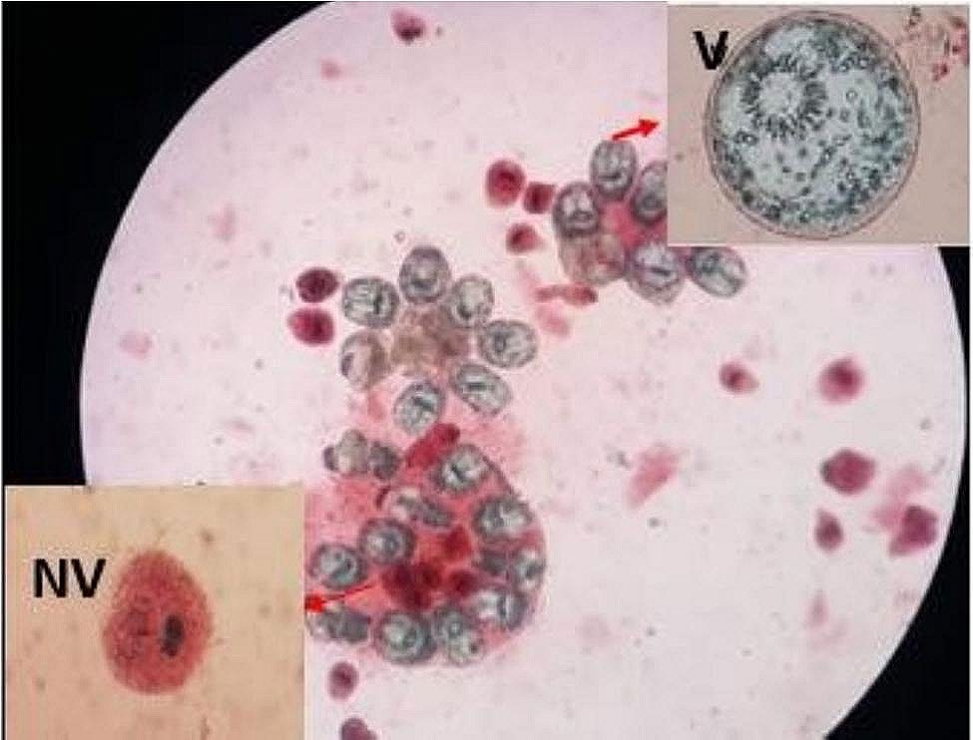
Direct observation at *10 magnification showing viable protoscolex (V) retaining their initial translucent color and non-viable protoscolex (NV) with total dye penetration after the 1‰ eosin colorimetric test

For the microscopic study, we performed a viability study of the protoscolex of echinococcal material. Indeed, 100 μl of the sedimentation pellet of the liquid, we added the same volume of eosin to 1‰ which was put between the slide and cover glass and let it act for 2 min, and then we observed under the light microscope at magnification × 10. Unstained protoscolex were viable and stained protoscolex were nonviable (Fig. 1). This examination allowed us to assess the presence of viable protoscolex within all protoscoleces and to calculate their percentage.

Data entry and analysis were performed using SPSS 20.0 software. The quantitative variables were described using the means, standard deviation, limits for variables with a Gaussian distribution, and in the opposite case, the median and extremes. The normality of the distribution of quantitative variables was studied using the Kolmogorov-Smirnov test. The qualitative variables were described using the calculation of the observed numbers and relative frequencies (percentages). We used the Chi-square test for the comparison of two or more frequencies, the Spearman correlation test for the study of the correlation between the quantitative variables, the t-test for the comparison of two averages when the conditions of application were verified and the Mann-Whitney test in the opposite case.

To determine the thresholds of the quantitative variables, we used the ROC curve (receiver operating characteristic). For the multivariate study, we used logistic regression using the 10% threshold for viability of echinococcal material. The significance level was set with an α risk at 5%.

## Results

During the study period, 27 (41%) of 66 operated LCE from 21 patients containing 248 DV were investigated in the Parasitology-Mycology laboratory. The average age of patients was 41 years with extremes ranging from 14 to 76 years. The most affected age group was between 30 and 39 years. Surgical history of the cystic echinococcosis was observed in only four patients (19%): two patients were operated on LCE, one patient for cystic echinococcosis of the lung, and one patient for cystic echinococcosis of the heart. Four patients (19%) were asymptomatic at the time of diagnosis. Pain was the main symptom and was found in 15 patients (71%). The other clinical signs were fever in seven patients (33%), cutaneous-mucosal jaundice in three patients (14%), acute cholangitis in four patients (19%). Liver function tests were pathological in 10 patients (47%). Eight patients (38%) presented with hepatic cholestasis. Hyperbilirubinemia was noted in five patients (62%). Hepatic cytolysis was noted in four patients (19%). 18 cysts (67%) were located in the right liver and nine (33%) in the left liver.

The median cyst size was 10 mm with extremes ranging from 37 to 170 mm. A size greater than 10 cm was found in 14 cysts (52%). 15 cysts (56%) were CE2 and contained 161 DV and 12 cysts containing 87 DV were CE3b. 15 cysts (55.5%) corresponding to 10 patients (47%) had imaging complications. Opening of the LCE into the bile duct was observed in 11 cysts (71%) and suppuration in four cysts (27%). In addition, no cyst rupture into the peritoneum or thorax was observed.

The macroscopic study of the EF at cyst puncture was rock water in nine cysts (33%), bilious in 10 cysts (37%), gelatinous in four patients (15%), purulent in four patients (15%). The median intracystic pressure was 43 mmHg (E: 9-152). The number of DV per cyst was variable. 15 cysts (57%) had a number of DV ≥ 10. The median size of the harvested DV was 1.56 (E: 0.1–4.24). The median DV volume was 2 mL (E: 0.1–40). For DV consistency, 173 DV (69.8%) were strained and 75 DV (30.2%) were flaccid. For wall transparency, 127 DV (51.2%) were opaque and 121 DV (48.8%) were translucent. Only one DV (0.4%) contained small daughter vesicles.

The viability rate of the protoscolex of the DV was 16.7% and that of the EF was 20%. The viability rate of EF from CE3b cysts was 9.2%, whereas that of CE2 cysts was 64%.

**Fig. 2:**
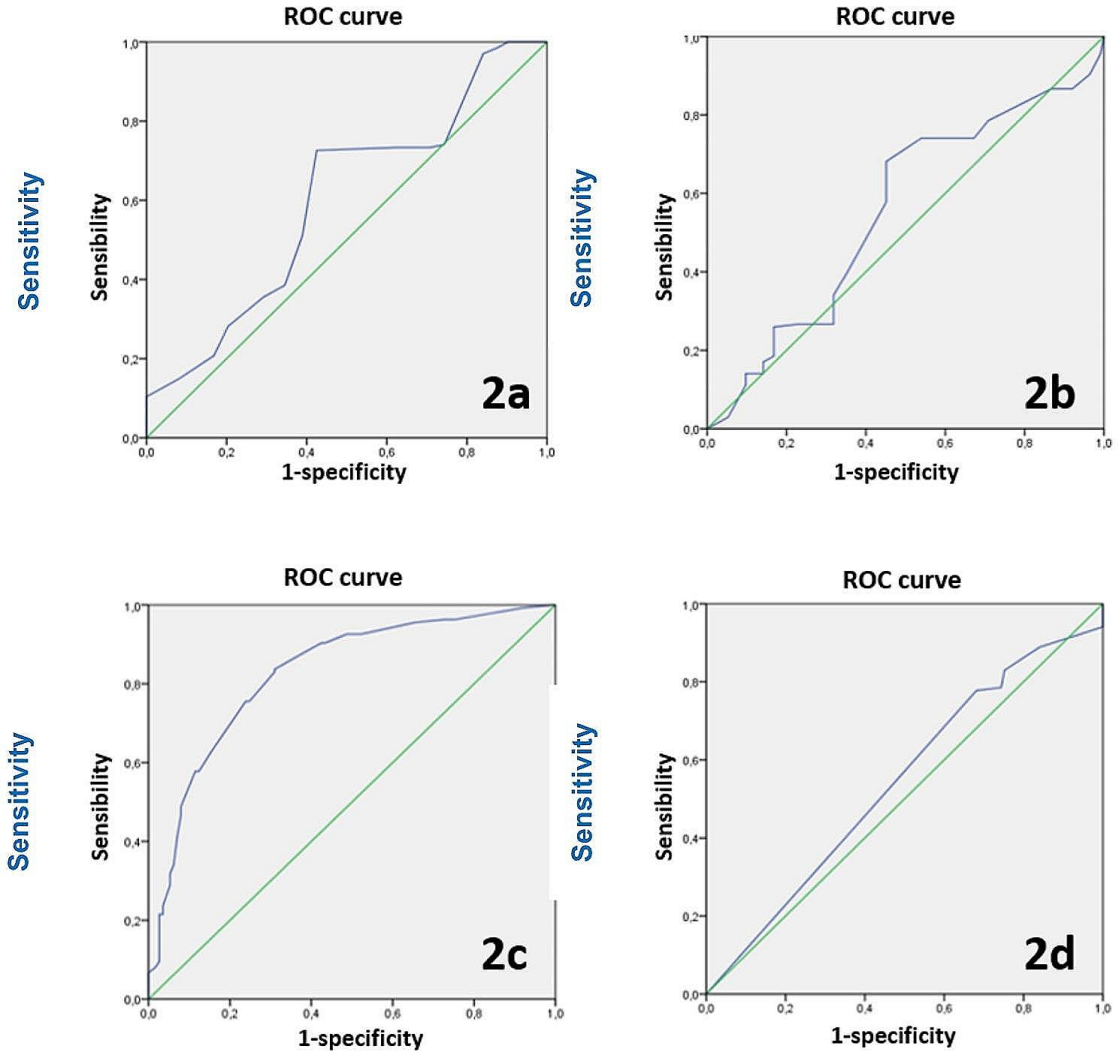
ROC curve: **2a**: cyst size/ **2b**: intracystic pressure/ **2c**: Daughter vesicle size/ **2d**: number of Daughter vesicle /cyst

For the analytical study of the daughter vesicles, in a bivariate study at the end of ours, fever, acute cholangitis, hyperbilirubinemia, localization in the left liver, rock water, bilious echinococcal fluid, the size of the cyst ≥ 43 mm, intracystic pressure ≥ 35 mmHg, DV size ≥ 6.5 mm, volume ≥ 0.15 ml, number of DV/cyst ≥ 5, and opaque wall were significantly associated with DV protoscolex viability. (Tables 1 and 2) The threshold for quantitative parameters (Table 3) is determined by the ROC curve (Fig. 2).

**Table 1:** Predictive factors for viability of DV protoscoleces in bivariate study (qualitative criteria)

Parameters	Correlation Coefficient, r	*p*
**Area**		
Age	-0.111	0.081
**Hepatic assessment**		
AST	-0.040	0.530
ALT	-0.118	0.063
ALP	-0.092	0.147
GGT	-0.025	0.7
*Total Bilirubin*	***0.308***	***< 0.001***
**Cyst characteristics**		
*Cyst size*	***0.254***	***< 0.001***
*Intra cystic pressure*	***0.167***	***0.008***
Peri-cyst thickness	-0.023	0.713
*Number of DV/Cysts*	***0.169***	***0.008***
EF Viability	0.017	0.089
**DV characteristics**		
DV Diameter	**0.557**	**< 0.001**
DV Volume	**0.557**	**< 0.001**

AST: aspartate aminotransferase; ALT: alanine aminotransferase; ALP: Alkaline phosphatase; GGT: Gamma-glutamyl transferase; DV: Daughter vesicle, EF: Echinococcal fluid

**Table 2:** Predictive factors of DV viability in bivariate study (qualitative criteria)

Parameters		Viability (%) Average (standard deviation)	*p*
**Area**			
Gender	Male (N = 143)	31.8 (35.3)	0.225
	Female (N = 105)	37.9 (40.8)	
Recurrent HEC	No (N = 222)	35.3 (37.9)	0.281
	Yes (N = 26)	26.8 (36)	
**Clinical signs**			
Pain	No (N = 74)	36.8 (41)	0.508
	Yes (N = 174)	33.3 (36.3)	
*Fever*	No (N = 173)	28.6 (35.1)	***< 0.001***
	***Yes (N = 75)***	***47.8 (40)***	
*Acute angiochilitis*	No (N = 220)	32.1 (37.7)	***0.007***
	***Yes (N = 28)***	***52.3 (33)***	
**Cyst characteristics**			
*WHO classification*	CE2	45.0 (39.5)	*< 0.001*
	***CE3b***	***14.8 (24.5)***	
*Located in liver*	***Left liver (N = 70)***	***43.2 (43.9)***	***0.038***
	Right Liver (N = 178)	30.9 (34.6)	
HF aspect			
*Rock water EF*	No (N = 150)	27.8 (32.9)	***0.001***
	***Yes (N = 98)***	***44.5 (42.4)***	
*Biliary EF*	No (N = 163)	30.1 (39.5)	***0.009***
	***Yes (N = 85)***	***42.6 (32.9***)	
*Purulent EF*	***No (N = 213)***	***39.3 (38)***	***< 0.001***
	Yes (N = 35)	4.3 (15.8)	
*Gelatinous EF*	***No (N = 218)***	***37.3 (38.4)***	***< 0.001***
	Yes(N = 30)	13.3 (24.8)	
**Complications**			
Cystobiliary fistula	No (N = 125)	33.7 (37)	0.318
	Yes (N = 123)	35.1 (38.6)	
*Suppurations*	***No (N = 218***)	***37.5 (38.4)***	***0.001***
	Yes (N = 30)	11.8 (23.1)	
**DV characteristics**			
Consistency	Strained (N = 173)	34.4 (36.6)	0.991
	Flaccid (N = 75)	34.4 (40.6)	
*Transparency*	***Opaque(N = 127)***	***39.2 (36.7)***	***0.042***
	Translucent (N = 121)	29.5 (38.4)	

HEC: hepatic echinococcal cyst; **EF**: Echinococcal fluid; DV: daughter vesicle

**Table 3:** Threshold for quantitative parameters according to ROC curves

Parameters	Threshold	Se (%)	Sp (%)	VPN (%)
Size of the cyst (Fig. 2a)	43 mm	100	10	100
	95 mm	72	58	63
	125 mm	35	80	48
Intracystic pressure (Fig. 2b)	9.5 mmHg	95	1	14
	35 mmHg	68	55	59
	61 mmHg	25	84	45
Size of the DV (Fig. 2c)	6.5 mm	99	8	100
	12.7 mm	83	70	72
	16.2 mm	60	89	66
Number of DV/cyst (Fig. 2d)	5	89	36	55
	7	71	40	51

Se: Sensitivity; Sp: specificity; VPN: negative predictive value; DV: daughter vesicle

CE3b type as classified by the WHO, purulent EF, and gelatinous EF were factors significantly associated with the non-viability of DV protoscoleces (Table 2).

However, other patient, cyst, and DV characteristics were not significantly associated with DV protoscolex viability.

In the multivariate study, at the end of ours, CE2 type as classified by WHO, bilirubinemia > 9.5 μmol/L, a number of DV/cyst ≥ 5, bilious echinococcal fluid, intracystic pressure ≥ 35 mmHg, DV volume > 0.15 ml, and DV size ≥ 6.5 mm were significantly associated with DV protoscolex viability (Table 4). Age and purulent echinococcal fluid were significantly associated with the non-viability of DV protoscoleces. (Table 4).

**Table 4:** Predictive factors of DV viability in multivariate study (all parameters included)

Predictive factors for the viability of DV protoscoleces	Odds Ratio	Confidence interval (95%)	*p*
Age (years)	0.8	0.5–1.1	0.001
WHO classification (CE2)	3.3	1.06–10.2	0.03
TB > 9.5 μmol/L	6.14	2.4–15.65	< 0.001
Number of DV /cyst ≥ 5	10.12	3.11–17.13	0.007
Biliary HF	5.63	2.44–13.29	< 0.001
Purulent EF purulent	0.13	0.02–0.66	0.014
Intracystic pressure ≥ 35 mmHg	8.5	3.3–21.37	< 0.001
DV volume **≥** 0.15 ml	11.31	5.14–24.9	< 0.001
DV size ≥ 6.5 mm	18.8	4.69–32.90	0.012

DV: daughter vesicle; TB: Total bilirubin; EF: Echinococcal fluid

Including only preoperative parameters, it was found that in multivariate analysis, CE2 type as classified by WHO, bilirubinemia > 9.5 μmol/L, cyst size ≥ 43 mm, number of DV/cyst ≥ 5, and DV size > 6.5 mm were significantly associated factors with DV protoscolex viability. Age was a factor significantly associated with the non-viability of DV protoscoleces. (Table 5)

**Table 5:** Predictive factors of DV viability in multivariate study (preoperative parameters)

Predictive factors for the viability of DV protoscoleces preoperatively		Odds Ratio	Confidence interval (95%)			*p*	
Age		0.5	0.2–0.7			0.003	
WHO classification (CE2)		22	18–38			0.001	
TB > 9.5 μmol/L		1.7	1.04–2.36			0.001	
Cyst size ≥ 43 mm		2.2	1.3–4.1			0.001	
Number of DV /cyst ≥ 5		2.7	3.11–17.13			0.05	
DV size ≥ 6.5 mm		11.4	5.8–17.1			0.001	

DV: Daughter Vesicle; TB: Total Bilirubin

## Discussion

At the end of this study, we found that the viability of the DV is associated with the size of the cyst, the number of DV per cyst, the size of the DV and intracystic pressure. This pressure reached the value of 152 mmHg in our series. Despite the strained consistency and pressure, the cyst did not rupture, which means that the wall of the parasite can resist rupture up to this limit. The non-viability of the DV is associated with CE3b type as classified by the WHO and age. Could these findings help us in the therapeutic management of the CE3b cyst?

There is a little work on the viability of protoscoleces. The results vary from one series to another due to the non-standardization of the methodologies applied to the study of parameters [1, 2, 4, 10–14]. These works are only interested in the study of echinococcal fluid from cysts in humans and animals.

Attached is a table of the different studies which were all retrospective. Only one prospective study on this subject was carried out by the team of El Saftawy A [14]. (Table 6) However, in the literature, no work has addressed the study of protoscolex viability within DV. Therefore, the management of cystic echinococcosis type CE3b (multivesicular cyst with mastic content) remains uncodified due to the lack of knowledge of the evolutionary process of DV. This is why this would be the first study that is interested in the content of the daughter vesicles of LCE which found that the DV can contain protoscolex which can be viable or non-viable.

**Table 6:** Summary table of the different studies analyzing the viability of protoscoleces for echinococcal fluid

	Year	Type of study	Number	Location of cyst	Viability
Tsimoyiannis EC, ^10^	2000	Retrospective	28	Liver (Human)	60%
Dueger E, ^11^	2001	Retrospective	212	Liver (animal)	73.2%
Monterola C, ^12^	2006	Retrospective	163	Liver (human)	42%
Bygott JM, ^4^	2010	Retrospective	63	Liver Lung (human)	62%
					70%
Zait H, ^1^	2013	Retrospective	78	Liver and lungs (human)	74%
El Saftawy A, ^14^	2021	Prospective	40	Liver (Human)	Viability according to the therapeutic protocol

To this day, the therapeutic strategy for LCE remains non-consensual [15] and is based on the characteristics of the cyst, particularly its activity, surgical expertise, the technical platform available, and the adherence of patients to long-term surveillance [16, 17].

While the therapeutic indications are increasingly codified for active cysts (I/II Gharbi and CE1/CE3a) candidate for aspiration or surgery and inactive cysts (IV/V Gharbi and CE4/CE5) [3–8] candidate for surveillance, those of cysts with transient activity, especially CE3b are not codified. Indeed, the 2 therapeutic modalities: surgery and surveillance: The ‘Wait and Watch’ procedure, can be proposed [3, 18, 19]. The results are contradictory in the literature and are not very precise. Some authors are in favor of the ‘Wait and Watch’ procedure, considering that these cysts with gelatinous contents have the same characteristics as the CE4 cyst, while others are in favor of surgery since they know nothing about the evolutionary process of the DV they contain.

In this study, we found that the predictive factors for non-viability of DV protoscoleces for the inherent preoperative parameters of the cyst and DV were age, CE3b type, cyst size < 4.3 cm, DV size < 6.5 mm, and number of DV/cyst < 5 with a statistically significant association (*p* = 0.003; 0.001; 0.001; 0.001, and 0.05, respectively). Thus, we could infer that gelatinous CE3b cysts with size < 4.3 cm with the largest DV size < 6.5 mm and a number of DV/cyst < 5 have inactive DV, and thus we could propose a ‘Wait and Watch’ procedure for these patients. Otherwise, for CE3b cysts with DV that do not have the criteria for inactivity, we could propose surgery.

Age was a factor significantly associated with non-viability of DV protoscoleces. This could be explained by the fact that the immune response may eventually surmount the parasite’s defense mechanisms over time and neutralize it. In fact, the parasite’s development relies on passive means and immunomodulation of the host’s immune system. These means may not be perfect or durable. So, this would constitute an argument in favor of the ‘Wait and Watch’ procedure.

However, a prospective study of the viability of echinococcal fluid from cysts essentially type CE3b with a calculation of the number of EF required is essential to complete our conclusions.

## Conclusion

The management of liver cystic echinococcosis requires a better understanding of aspects related to the survival of the parasite, particularly protoscolex. Both daughter vesicles and echinococcal fluid from cysts may be viable. The predictive factors for viability of daughter vesicles are cyst size ≥ 43 mm, DV size ≥ 6.5 mm, number of DV per cyst ≥ 5, and WHO CE2 type. CE3b and age are predictive factors for the non-viability of DV. CE3b cysts with DV that do not meet these criteria may be candidates for a surveillance or ‘Wait and Watch’ procedure.

## Data Availability

The datasets used and/or analyzed during the current study available from the corresponding author on reasonable request. Aymen Trigui (ayman.trigui@gmail.com).
